# Evaluating an advanced double intravenous vasopressor automated system to treat hypotension during spinal anesthesia for cesarean delivery: a randomized controlled trial

**DOI:** 10.1186/s12871-023-01992-7

**Published:** 2023-01-26

**Authors:** Hon Sen Tan, Singaraselvan Nagarajan, Jason Ju In Chan, Chin Wen Tan, Rehena Sultana, Alex Tiong Heng Sia, Ban Leong Sng

**Affiliations:** 1 Department of Women’s Anesthesia, KK Women’s and Children’s Hospital, Singapore, Singapore; 2 Anesthesiology and Perioperative Sciences Academic Clinical Program, Duke-NUS Medical School, Singapore, Singapore; 3 Center for Quantitative Medicine, Duke-NUS Medical School, Singapore, Singapore

**Keywords:** Anesthesia, Cesarean delivery, Ephedrine, Hemodynamics, Hypotension, Phenylephrine

## Abstract

**Background:**

The optimal treatment of hypotension during spinal anaesthesia is uncertain. A novel double intravenous vasopressor automated (DIVA) system reduces hypotension compared to standard care, and was subsequently modified to an advanced-DIVA (ADIVA) system. The primary objective was to compare ADIVA versus DIVA on incidence of hypotension (systolic BP (SBP) < 80% baseline).

**Methods:**

We conducted a randomized-controlled trial in women undergoing elective cesarean delivery under spinal anesthesia. SBP and heart rate were measured continuously using a Nexfin monitor. ADIVA delivered 25 μg phenylephrine (heart rate > 60 beats.min^−1^) or 2 mg ephedrine (heart rate < 60 beats.min^−1^) at SBP 90 to 110% of baseline, 50 μg phenylephrine or 4 mg ephedrine at SBP 80 to 90%, and 75 μg phenylephrine or 6 mg ephedrine at SBP < 80%. ADIVA calculated the trend of SBP; vasopressors were administered rapidly if SBP trended downward, or 30 s if SBP trended upward. In contrast, DIVA delivered 25 μg phenylephrine or 2 mg ephedrine at SBP 90 to 100% of baseline, and 50 μg phenylephrine or 4 mg ephedrine at SBP < 90%. Boluses were followed by a 10-s lockout. Other outcomes included hypertension (SBP > 120% baseline), vasopressor consumption, clinical outcomes, and performance measures from spinal anesthesia to fetal delivery.

**Results:**

We analyzed 94 parturients (ADIVA: *n* = 46, DIVA: *n* = 48), with no difference in the incidence of hypotension between ADIVA (78.3%) and DIVA (83.3%, *p* = 0.677). ADIVA had significantly higher proportion of hypotensive SBP readings, lower phenylephrine consumption and higher umbilical arterial pH. There was no difference in hypertension, bradycardia, ephedrine consumption, intravenous fluid volume, nausea/vomiting, Apgar scores, and umbilical venous pH or lactate. ADIVA maintained SBP higher above baseline with greater fluctuation than DIVA.

**Conclusion:**

ADIVA was associated with a greater proportion of hypotensive SBP readings, reduced phenylephrine consumption, and increased umbilical arterial pH than DIVA. Further research is needed to determine the optimal method of vasopressor delivery in parturients undergoing cesarean delivery.

**Trial registration:**

This study was registered on Clinicaltrials.gov registry (NCT03620942) on 08/08/2018.

## Introduction

Hypotension occurs in up to 80% of women during cesarean delivery under spinal anesthesia [1]. Given that cesarean delivery accounts for 21% of all deliveries worldwide [2], a large population is at risk of hypotension-related morbidity including nausea, vomiting, and fetal acidosis [3–5]. Reducing hypotension during cesarean delivery is an important goal within clinical guidelines and enhancing recovery after cesarean delivery protocols, with significant healthcare resources allocated to its prevention and management [3, 6].

The optimum method of preventing or treating hypotension during cesarean delivery is uncertain, although vasopressors have demonstrated efficacy and are mainstays of contemporary clinical practice [7]. Vasopressors such as phenylephrine and ephedrine are commonly administered to treat hypotension after it has occurred, however, such reactive treatment may be associated with significant lag time before baseline blood pressure is restored. This lag may arise from delay in detecting hypotension as standard intermittent oscillometric blood pressure monitors are unable to detect rapid blood pressure changes [8]. Furthermore, wide inter-individual variability in the severity of hypotension and response to vasopressor treatment often lead to under- or over treatment, with the latter associated with complications such as reactive hypertension and cardiac arrhythmias [7]. In short, one of the key barriers preventing optimal management of hypotension is our inability to detect hypotension and administer titrated doses of vasopressors in a timely manner.

We have previously described a novel double intravenous vasopressor automated (DIVA) closed-loop system that analyzed beat-to-beat systolic blood pressure data (SBP) from a continuous non-invasive hemodynamic monitor (Nexfin, BMEYE, B.V., Amsterdam) [9]. DIVA achieved lower incidence of hypotension and less wobble, with no significant difference in the incidence of nausea, vomiting, reactive hypertension, or vasopressor doses compared to standard care [10]. However, despite the efficacy of DIVA, almost 40% of parturients experienced one or more episodes of hypotension during cesarean delivery [10], suggesting that further refinement is required. We subsequently developed the advanced-DIVA (ADIVA) system that determined if SBP was trending up or down in the ten seconds prior to vasopressor delivery. If ADIVA detected a downward SBP trend, vasopressors were administered rapidly (between 4.5 to 13.5 s depending on dose), conversely, vasopressors were delivered over 30 s if SBP was trending upwards [8]. Delivering vasopressors rapidly when SBP was falling may help ADIVA to rapidly return SBP to baseline levels and forestall hypotension, while slow infusion of vasopressors when SBP was rising may reduce the incidence of reactive hypertension. The aim of this randomized controlled trial was to evaluate the efficacy of ADIVA compared to DIVA, with the primary outcome being incidence of hypotension defined as any SBP reading < 80% of baseline from spinal anesthesia to fetal delivery. Secondary outcomes included the incidence of hypertension and bradycardia, vasopressor consumption, incidence of nausea and vomiting, umbilical pH and Apgar scores, and system performance measures.

## Methods

This randomized controlled study was conducted from January 2020 to September 2021 at KK Women’s and Children’s Hospital, Singapore, after approval by the SingHealth Centralized Institutional Review Board (Ref: 2018/2213) on 06/04/2018 and registration on clinicaltrials.gov (NCT03620942) on 08/08/2018. Written informed consent was obtained from all participants in accordance with the Declaration of Helsinki, and this manuscript adhered to the relevant Consolidated Standards of Reporting Trials (CONSORT) guidelines.

We included parturients aged 21 to 45 years old, weighing 40 to 100 kg, 145 to 170 cm in height, American Society of Anesthesiologists (ASA) physical status 2, with singleton full term pregnancies undergoing spinal anesthesia for elective cesarean delivery. Parturients with contraindication to spinal anesthesia, hypertensive disorders requiring medication, premature rupture of amniotic membranes for > 48 h, diabetes requiring insulin, and uncontrolled medical conditions such as cardiac disease were excluded.

Parturients were randomized (1:1 ratio) to receive ADIVA or DIVA using a computer-generated random number generator. The allocation sequence was created by the study statistician and concealed using sequentially numbered opaque sealed envelopes. Prior to spinal anesthesia, the study investigator opened the envelope containing the group allocation. Parturients, obstetricians, nurses, and anesthesiologists involved in anesthesia management and data collection were blinded to the group allocation.

Baseline SBP was measured in the ward as the mean of three consecutive readings taken at one-minute intervals using an oscillometric device on the right arm with the patient supine and left uterine displacement. Intravenous access was obtained using an 18-gauge cannula, pulse oximeter and electrocardiogram were applied, and the Nexfin finger cuff was attached to the right second or middle finger. Spinal anesthesia was performed in the sitting flexed position with a 27-gauge Whitacre needle (BD Medical, New Jersey, USA), 11 mg hyperbaric bupivacaine 0.5%, 15 μg fentanyl, and 100 μg morphine. The parturient was then positioned supine with left uterine displacement, free flowing infusion of lactated ringers solution was commenced, and the ADIVA system was initiated by the study investigator.

**Fig. 1:**
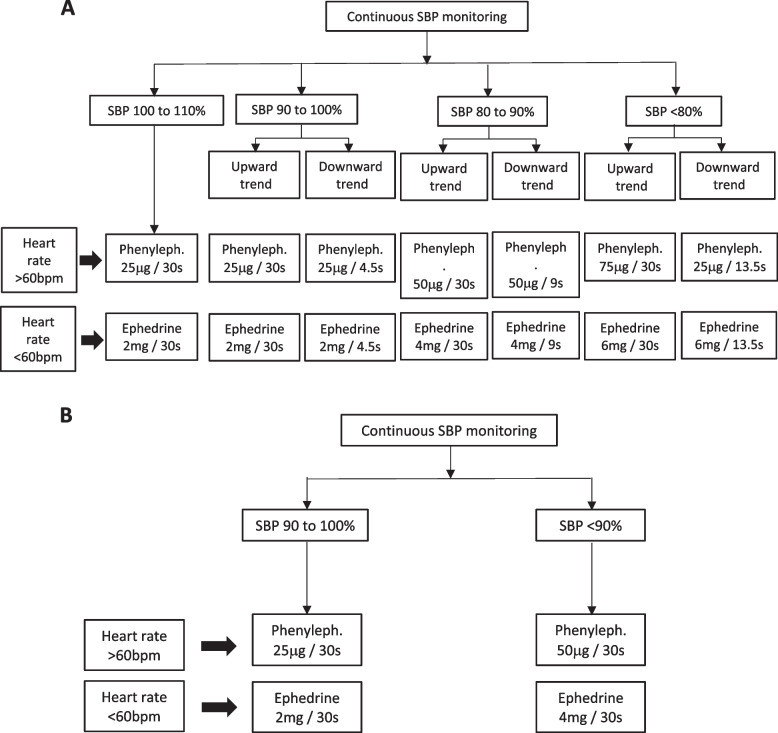
Schematic diagram of the algorithm used in (**A**) ADIVA, and (**B**) DIVA. ADIVA: Advanced double intravenous vasopressor automated; DIVA: double intravenous vasopressor automated

SBP and heart rate measurements by Nexfin were uploaded continuously to a laptop computer which integrated the data every 10 s to form a moving average value (LabVIEW running on Windows XP, Microsoft Corporation, Washington, USA). The drug delivery system consisted of two syringe driver pumps (B.Braun, Melsungen, Germany) with one 50 ml syringe filled with 100 μg.ml^−1^ phenylephrine and another 50 ml syringe containing 8 mg.ml^−1^ ephedrine, both connected to the intravenous cannula by three-way stopcocks. Parturients randomized to the ADIVA system (Fig. 1A) received 25 μg phenylephrine (heart rate > 60 beats.min^−1^) or 2 mg ephedrine (heart rate < 60 beats.min^−1^) if SBP fell between 90 to 110% of baseline, 50 μg phenylephrine or 4 mg ephedrine if SBP was between 80 to 90% of baseline, and 75 μg phenylephrine or 6 mg ephedrine if SBP dropped below 80% of baseline. ADIVA calculated the moving average SBP every ten seconds, and the gradient of the slope of SBP changes was calculated using the least squares method. Vasopressors were administered rapidly (over 4.5 to 13.5 s depending on vasopressor dose) if SBP trended downward in the ten-second interval preceding vasopressor delivery, while the same vasopressor dose was delivered over 30 s if SBP trended upward [8]. Conversely, parturients randomized to the DIVA system received 25 μg phenylephrine (heart rate > 60 beats.min^−1^) or 2 mg ephedrine (heart rate < 60 beats.min^−1^) if SBP fell between 90 to 100% of baseline, and 50 μg phenylephrine or 4 mg ephedrine if SBP dropped below 90% of baseline (Fig. 1B). A lockout period of 10 s occurred after each vasopressor bolus in both ADIVA and DIVA systems. Both systems were monitored by an investigator who could manually administer atropine and/or vasopressors in the event of bradycardia or if SBP remained < 70% of baseline for more than three minutes.

Five minutes after spinal anesthesia, sensory block was assessed using loss of sensation to cold while motor block was measured using the modified Bromage scale [11]. Patient and clinical characteristics, SBP, heart rate, duration of spinal anesthesia to fetal delivery, vasopressor doses, incidence of nausea/vomiting, umbilical cord pH, and Apgar scores were recorded. We evaluated ADIVA and DIVA using performance measures previously employed on similar closed-loop systems, adjusted with a pooled-data approach that provides consideration for the wide variation in number of measurements taken for each parturient, described below [12–14].

### Percentage performance error (PE)

Percentage performance error was defined as the percentage difference between each SBP value from the baseline, and is calculated for the i^th^ parturient at the j^th^ second as follows:

$$PEij=\frac{Measured\;BPij-Baseline\;BPi}{Baseline\;BPi}\;X\;100$$

### Median absolute performance error (MDAPE)

MDAPE indicates the absolute magnitude of differences between measured and baseline SBP, and is a measure of inaccuracy. It is defined as the median of absolute PE (|PE|) values, calculated as follows where N_i_ is the number of values of |PE| for the i^th^ parturient and M is the number of parturients in the study:

$$MDAPEi=median \{\left|PEij\right|, j=1, \dots , Ni\}$$

$$MDAPE=\frac1{\sum_{i=1}^MNi}X\sum_{i=1}^M(Ni\;X\;MDAPEi)$$

### Median performance error (MDPE)

MDPE is a measure of bias, and indicates whether SBP was systematically above or below the baseline, calculated as follows:

$$MDPEi=median \{PEij, j=1, \dots , Ni\}$$

$$MDPE=\frac1{\sum_{i=1}^MNi}\;X\;\sum_{i=1}^M(NiXMDPEi)$$

### Wobble

Wobble measures how much PE fluctuates around the MDPE with time for each parturient, calculated as follows:

$$WOBBLEi=median\;\{\vert PEij-MDPEi\vert,j=1,\dots,Ni\}$$

$$WOBBLE=\frac1{\sum_{i=1}^MNi}\;X\overset M{\underset{i=1}{\;\sum}}(Ni\;X\;WOBBLEi)$$

### Divergence

Divergence is the slope obtained from linear regression of each parturient’s |PE| with time, and assesses the trend of |PE| change over time, thereby indicating if the system accuracy is improving (negative divergence) or worsening (positive divergence) with time. Divergence (per minute) was calculated as follows where t_ij_ is the time of i^th^ individual measurement in minutes:

$$DIVERGENCEi=\frac{\sum_{j=1}^{Ni}\left|PEij\right|\;X\;tij-(\sum_{J=1}^{Ni}PEij)X\left(\sum_{j=1}^{Ni}tij\right)/Ni}{{\sum_{j=1}^{Ni}\left(tij\right)}^2={(\sum_{j=1}^{Ni}tij)}^2/Ni}$$

$$DIVERGENCE=\frac1{\sum_{i=1}^MNi}\;X\;\sum_{i=1}^M(Ni\;X\;DIVERGENCEi)$$

### Statistical analyses

Continuous and categorical variables were summarized as mean ± standard deviation (SD) or median (interquartile range (IQR) [range]) as appropriate, or number (proportion) respectively. Categorical and continuous variables were compared using the X^2^ test, two-sample t-test, or Mann–Whitney U-test as appropriate. All analyses were performed using SAS version 9.4 (SAS Institute, North Carolina, USA).

A sample size of 92 parturients (46 in each group) is required to detect 25.5% absolute difference in the incidence of hypotension, based on the following assumptions: incidence of hypotension in ADIVA group of 13.5%, incidence of hypotension in DIVA group of 39.0% based on previous DIVA studies on reported incidence and difference in incidence [10, 14], 1:1 allocation ratio, alpha of 5%, power of 80%, and 5% loss due to failure of spinal anesthesia.

## Results

**Fig. 2:**
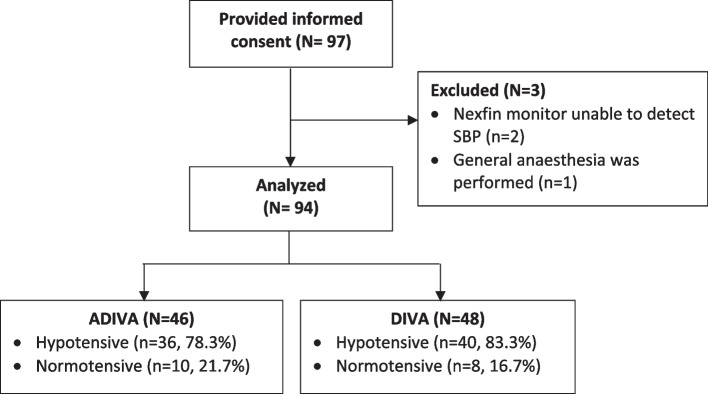
Study flow diagram

We enrolled 97 parturients in this study, and analyzed data from 94 (ADIVA: 46, DIVA: 48) after exclusion of three parturients (Fig. 2). There was no significant difference in baseline parturient and surgical characteristics (Table 1). Of note, sensory block height and duration from spinal anesthesia to fetal delivery were similar in both groups.

**Table 1:** Parturient and surgical characteristics

Variable	ADIVA (*n* = 46)	DIVA (*n* = 48)	*p*-value
Age (years)	33.7 ± 4.3	33.0 ± 4.2	0.429
Weight (kg)	72.8 ± 12.8	70.8 ± 9.4	0.409
Height (cm)	157.8 ± 6.2	158.9 ± 5.3	0.366
BMI (kg cm^−2^)	29.2 ± 4.8	28.1 ± 3.6	0.200
Race			0.132
Chinese	22 (47.8)	28 (58.3)	
Malay	10 (21.7)	6 (12.5)	
Indian	2 (4.3)	7 (14.6)	
Others	12 (26.1)	7 (14.6)	
Gestational age (weeks)	38.6 ± 0.8	38.8 ± 0.8	0.094
Baseline systolic blood pressure (mmHg)	108.9 ± 9.7	107.6 ± 9.6	0.522
Sensory block dermatome			0.087
T3	2 (4.4)	0 (0.0)	
T4	39 (86.7)	46 (97.9)	
T5	4 (8.9)	1 (2.1)	
Spinal anesthesia to delivery (mins)	21.0 (12.5) [9 to 588]	21.0 (8.0) [12 to 622]	0.176
Spinal anesthesia to end of surgery (mins)	63.5 (51.0) [30 to 697]	68.5 (45.0) [35 to 668]	0.112

Values are expressed in mean ± standard deviation (SD) or median (interquartile range (IQR) [range]) or number (%)

*ADIVA* Advanced double intravenous vasopressor automated, *BMI* body mass index, *DIVA* double intravenous vasopressor automated

Clinical and hemodynamic outcomes are summarized in Table 2. Of 94 parturients, 76 (80.9%) developed hypotension, with no significant difference between parturients who received ADIVA (*n* = 36, 78.3%) versus DIVA (*n* = 40, 83.3%, *p* = 0.677). ADIVA was associated with lower proportion of SBP readings between 80 to 120% of baseline, and higher proportion of SBP readings below 80% of baseline compared to DIVA, but there was no significant difference in the incidence of hypertension and minimum and maximum SBP. Parturients receiving ADIVA had significantly lower phenylephrine consumption, but no significant difference in the incidence of bradycardia, minimum and maximum heart rates, ephedrine consumption, and volume of intravenous fluids compared to those in the DIVA group. Likewise, ADIVA was associated with significantly higher umbilical arterial pH, but other clinical outcomes such as the incidence of nausea or vomiting, fetal weight and Apgar scores, umbilical venous pH, and umbilical artery and lactate were comparable with DIVA.

**Table 2:** Clinical and hemodynamic outcomes

Variable	ADIVA (*n* = 46)	DIVA (*n* = 48)	*p*-value
Incidence of hypotension ^a,b^	36 (78.3)	40 (83.3)	0.677
Incidence of hypertension ^a,c^	43 (93.5)	40 (83.3)	0.199
Incidence of bradycardia ^a,d^	0 (0.0)	0 (0.0)	-
Minimum SBP (mmHg) ^a^	69.8 ± 21.0	69.2 ± 17.0	0.881
Maximum SBP (mmHg) ^a^	163.4 ± 46.7	154.4 ± 41.6	0.323
SBP readings within 80% to 120% of baseline ^a^	93,432/125,966 (74.2)	109,458/128,991 (84.9)	< 0.001
SBP readings below 80% of baseline ^a^	11,105/125,966 (8.8)	7460/128,991 (5.8)	< 0.001
Minimum heart rate (beats.min^−1^) ^a^	51.2 ± 13.9	54.3 ± 14.8	0.300
Maximum heart rate (beats.min^−1^) ^a^	115.9 ± 14.9	116.7 ± 17.4	0.812
Total phenylephrine dose (μg) ^a^	100 (150) [0 to 400]	150 (250) [0 to 1150]	0.014
Total ephedrine dose (mg) ^a^	0 (0) [0 to 16]	0 (0) [0 to 8]	0.504
Total intravenous fluid volume (ml) ^a^	500 (300) [200 to 1000]	550 (100) [150 to 1500]	0.229
Incidence of nausea ^a^	7 (15.2)	10 (20.8)	0.595
Incidence of vomiting ^a^	6 (13.0)	8 (16.7)	0.774
Fetal weight (g)	3200 ± 340	3100 ± 360	0.679
Apgar score (1 min)	9 [8 to 9]	9 [8 to 9]	1.000
Apgar score (5 min)	9 [9 to 9]	9 [9 to 9]	–
Umbilical arterial pH	6.9 ± 1.5	5.8 ± 3.0	0.019
Umbilical venous pH	7.2 ± 1.1	7.2 ± 1.1	0.955
Umbilical arterial lactate	2.4 ± 0.9	2.1 ± 1.4	0.157
Umbilical venous lactate	1.7 ± 0.5	1.8 ± 0.5	0.441

Values are expressed in mean ± standard deviation (SD) or median (interquartile range (IQR) [range]) or number (%)

*ADIVA* Advanced double intravenous vasopressor automated, *DIVA* Double intravenous vasopressor automated, *SBP* Systolic blood pressure

^a^ During the period from spinal anesthesia to fetal delivery

^b^ Defined as any SBP reading below 80% of baseline

^c^ Defined as any SBP reading above 120% of baseline

^d^ Defined as any heart rate reading below 60 beats.min^−1^

In terms of closed-loop system performance, both ADIVA exhibited significantly greater MDAPE and wobble compared to DIVA, while MDPE and divergence were similar between the two systems (Table 3).

**Table 3:** Performance measures of ADIVA and DIVA closed-loop systems

Variable	ADIVA (*n* = 46)	DIVA (*n* = 48)	*p*-value
Median absolute performance error (%)	13.1 ± 5.2	9.5 ± 4.1	0.001
Median performance error (%)	5.4 ± 10.9	3.2 ± 8.5	0.276
Wobble (%)	8.3 ± 3.5	6.3 ± 2.5	0.002
Divergence (%.min^−1^)	-0.1 ± 0.3	-0.1 ± 0.4	0.955

Values are expressed in mean ± standard deviation (SD)

*ADIVA* Advanced double intravenous vasopressor automated, *DIVA* Double intravenous vasopressor automated

## Discussion

In this randomized controlled study, we compared the performance of ADIVA versus DIVA in managing hypotension during spinal anesthesia for cesarean delivery. The use of ADIVA did not significantly change the incidence of hypotension, hypertension, bradycardia, ephedrine consumption, and other relevant clinical outcomes compared to DIVA. However, ADIVA was associated with a greater proportion of hypotensive SBP readings, reduced phenylephrine consumption, and increased umbilical arterial pH than DIVA. Lastly, ADIVA exhibited greater MDAPE and wobble than DIVA, although MDPE and divergence were comparable between the two systems.

Three modifications were incorporated into ADIVA that we postulated will improve hemodynamic stability. First, ADIVA delivered low vasopressor doses at SBP was between 90 to 110% of baseline, in contrast to DIVA which administered the same vasopressor dose when SBP fell between 90 to 100% of baseline. We hypothesized that pre-emptive use of small vasopressor doses may prevent SBP from dropping below baseline levels and reduce the incidence of hypotension, albeit at the theoretical cost of increased hypertension risk. However, we did not detect a significant difference in the incidence of hypotension and hypertension between ADIVA and DIVA, which may suggest that the vasopressor dose (25 μg phenylephrine or 2 mg ephedrine) was insufficient to forestall the development of hypotension. Future studies should investigate if a higher pre-emptive vasopressor dose delivered between 90 to 110% of baseline reduces the incidence of hypotension, without concomitant increase in the risk of hypertension.

Second, hypotensive parturients with SBP readings below 80% of baseline received larger vasopressor doses (75 μg phenylephrine or 6 mg ephedrine) from ADIVA, in contrast to the 50 μg phenylephrine or 4 mg ephedrine administered by DIVA. We postulated that larger vasopressor doses will rapidly return SBP above 80% of baseline and minimize the duration of hypotension, but paradoxically, our results showed that ADIVA had greater proportion of hypotensive SBP readings compared to DIVA. Given that SBP was monitored continuously in a beat-to-beat fashion, it is possible that short periods of hypotension occurring during vasopressor administration or the subsequent 10 s lockout period were not treated, hence preventing higher vasopressor doses from reducing the proportion of hypotensive readings.

Third, ADIVA calculated the SBP trend and administered vasopressors rapidly if SBP trended downward in the ten-second interval preceding vasopressor delivery, while the same vasopressor dose would be delivered over 30 s if SBP trended upward. In comparison, DIVA delivered all vasopressors over 30 s, regardless of SBP trend. We hypothesized that delivering vasopressors rapidly may halt the decline in SBP and hence forestall the onset of hypotension, while slow infusion of vasopressors with rising SBP may reduce the incidence of hypertension. However, our results showed that this was not the case, as no significant difference in the incidence of hypotension was found between ADIVA and DIVA groups.

When evaluated with performance measures used to evaluate similar closed-loop systems, ADIVA exhibited significantly higher MDAPE of 13.1% compared to DIVA (9.5%), indicating that ADIVA resulted in greater deviation of SBP values above and below the baseline. Also, ADIVA was associated with a MDPE of 5.4%, showing that SBP values were maintained at a median of 5.4% above baseline, compared to 3.2% with DIVA. Similarly, wobble was higher in ADIVA (8.3%) than DIVA (6.3%), indicating that the former was associated with greater fluctuation of SBP values around MDPE. The combination of these findings suggests that ADIVA tended to maintain SBP values higher above baseline with greater SBP fluctuation than DIVA. This may be due to the addition of pre-emptive vasopressor delivery starting at 110% of baseline, as well as the rapid administration of vasopressors that may introduce greater fluctuation in SBP readings.

It should be noted that the incidence of hypotension in this study was markedly greater than in our previous randomized controlled study comparing DIVA with manual vasopressor boluses [10], although it was comparable to our pilot study of ADIVA [8]. This may be due to the difference in intervals at which SBP was measured; in the study comparing DIVA with manual boluses, SBP was measured at one-minute intervals, while it was measured continuously in the ADIVA pilot study. Intermittent measurement of SBP may omit short periods of hypotension, which would have been recorded if SBP was measured continuously.

The use of a closed-loop feedback system for vasopressor delivery was firstly described by Ngan Kee et al. by maintaining the maternal blood pressure in Cesarean patients with a simple on–off algorithm to activate phenylephrine infusion of 100 μg/min [13]. The same group later utilized a closed-loop feedback computer-controlled system with variable phenylephrine infusion regimen to deliver phenylephrine at infusion rates ranging from 0 to 100 μg/min. The group compared the novel algorithm with the predecessor on–off algorithm; and showed that the former was associated with fewer physician-guided intervention with superior stability in hemodynamic parameters [15]. This was follow-up with a randomized controlled trial by modifying the method of phenylephrine administration from continuous infusion to intermittent boluses, however the findings showed no significant difference in clinical outcomes even though the precision of blood pressure control was greater when phenylephrine was delivered using intermittent boluses as compared with continuous infusion [16]. Other research groups also investigated the use of automated closed-loop systems for vasopressor delivery for hemodynamic management and showed similar performances as physician-guided intervention [17, 18]; however these systems are only tested in animal models and therefore future studies are warranted to refine these novel systems and to test them in clinical context.

We acknowledge several limitations in this study. Our study may have been inadequately powered to detect small differences in the incidence of hypotension between parturients receiving ADIVA compared to those receiving DIVA, although the clinical significance of such differences is debatable. In this study, we chose to use a validated non-invasive blood pressure monitoring, Nexfin, rather than intra-arterial measurement which is invasive with accompanying risk of adverse events. The accuracy and precision of Nexfin for continuous SBP measurement may be affected by movement or cold temperature leading to peripheral vasoconstriction, nonetheless, the accuracy of Nexfin has been validated against intra-arterial measurements [19]. Finally, the cost and complexity of ADIVA and DIVA systems limit their generalizability and widespread adoption.

## Conclusion

ADIVA did not significantly improve hemodynamic control and clinical outcomes during spinal anesthesia compared to DIVA. However, ADIVA was associated with a greater proportion of hypotensive SBP readings, reduced phenylephrine consumption, and increased umbilical arterial pH than DIVA. Further research is needed to determine the optimal method of vasopressor delivery in parturients undergoing cesarean delivery.

## Data Availability

The datasets generated and/or analyzed during this study are not publicly available due to institutional policy on data confidentiality but are available from the corresponding author on reasonable request.

## Abbreviations

ASA: American Society of Anesthesiologists
ADIVA: Advanced double intravenous vasopressor automated
BMI: Body mass index
CONSORT: Consolidated Standards of Reporting Trials
DIVA: Double intravenous vasopressor automated
MDAPE: Median absolute performance error
MDPE: Median performance error
PE: Performance error
SBP: Systolic blood pressure
SD: Standard deviation
